# Renoprotective Effects of a New Free Radical Scavenger, XH-003, against Cisplatin-Induced Nephrotoxicity

**DOI:** 10.1155/2020/9820168

**Published:** 2020-04-18

**Authors:** Ya-Hong Liu, Kui Li, Hong-Qi Tian

**Affiliations:** Tianjin Key Laboratory of Radiation Medicine and Molecular Nuclear Medicine, Institute of Radiation Medicine, Chinese Academy of Medical Sciences and Peking Union Medical College, No. 238, Baidi Road, Tianjin, China

## Abstract

Acute renal injury has an incidence of 25%–30% in patients with tumors who are treated with cisplatin and in patients for whom no specific drugs are available for treatment. Amifostine is the only FDA-approved chemoprotective drug; however, its clinical application is limited because of side effects. The small-molecule antioxidant XH-003, an acute radiation syndrome- (ARS-) protective drug independently developed in our laboratory, with 100% intellectual property rights, overcomes the side effects of amifostine but retains its high efficacy. In this study, XH-003 showed a chemoprotective effect similar to that of amifostine. A mechanistic study showed that XH-003 could significantly reduce cisplatin-induced increases in serum creatinine and urea nitrogen, increase the activity of antioxidant enzymes (SOD, CAT, and GSH-Px), reduce oxidative stress and tissue inflammation, and alleviate renal tissue damage by blocking the activity of the mitochondrial apoptosis pathway. Most importantly, XH-003 could reduce the accumulation of cisplatin in renal tissue by regulating the expression of proteins involved in cisplatin uptake and excretion, such as organic cation transporter 2 and MRP2. Moreover, in an *in vivo* xenotransplantation model, XH-003 did not interfere with the antitumor effect of cisplatin. These data provide strong evidence that the ARS-protective agent has a great potential for protecting against chemotherapy-induced toxicity. Thus, XH-003 can be considered in antitumor therapy.

## 1. Introduction

Cisplatin (DDP), a potent chemotherapeutic agent, is widely used to treat various types of solid tumors, such as bladder, cervical, head and neck, esophageal, triple-negative breast, and small-cell lung cancers [1–3]; however, it has severe side effects including ototoxicity, neurotoxicity, and nephrotoxicity. Mounting clinical evidence has shown that acute kidney injury (AKI) is developed in approximately 25%–30% of patients treated with DDP [4]. AKI is associated with preferential accumulation of DDP in renal tubules, resulting in renal dysfunction [5]. However, the detailed mechanism of DDP-induced AKI remains elusive.

The proposed pathophysiological mechanisms of DDP-induced nephrotoxicity primarily involve DNA damage, the mitochondrial apoptosis pathway, inflammation, and oxidative stress [6–8]. The uptake of DDP by renal tubular epithelial cells involves organic cation transporter 2 (OCT2) and copper transporter 1 (CTR1). After entering cells, the chlorine atom of DDP is replaced by water by hydration. Therefore, electrophilic compounds produced by DDP can interact with nuclear DNA and activate the p53 protein. DDP can also interact with mitochondrial DNA, reduce the expression of electron transport chain proteins, damage respiration, and increase the production of reactive oxygen species (ROS). ROS, in turn, can induce oxidative stress and activate p53, which ultimately activates the apoptotic pathway. The increase in ROS can also induce proinflammatory factors, resulting in inflammation. In general, DDP-enhanced ROS production is the key contributor to renal dysfunction. Therefore, inhibition of ROS by antioxidants is a potential approach to the treatment of DDP-induced nephrotoxicity [9].

Amifostine [10] (Ethyol®), a highly efficient ROS scavenger, has been developed by the Walter Reed Army Institute in 1959 as an acute radiation syndrome- (ARS-) protective agent for soldiers in the Cold War and has been approved by FDA for the reduction of cumulative renal toxicity associated with repeated administration of DDP in patients with advanced ovarian cancer in 1995. However, owing to its short half-life, injection-only administration, strong side effects (nausea, vomiting, hypotension, etc.), and poor patient compliance, the clinical application of amifostine is limited [10]. At present, hydration and diuresis are primarily used to protect against DDP-induced nephrotoxicity [11] by reducing the concentration of DDP in renal tubules and by reducing renal damage. However, this method requires consumption of large volumes of water, resulting in frequent urination, which is inconvenient for patients. Meanwhile, the guidance on DDP hydration requires improvements. More importantly, hydration and diuresis do not protect from renal dysfunction in a percentage of treated patients. In addition, in the primary stage, researches have reported that natural antioxidants, such as capsaicin [12, 13], curcumin [14–16], ellagic acid [17–19], epigallocatechin-3-*O*-gallate [20–22], *α*-lipoic acid [23, 24], lycopene [25, 26], quercetin [27, 28], resveratrol [29, 30], sulforaphane [31, 32], tannic acid [33, 34], and vitamins [35–39], can alleviate the DDP-induced increase in serum creatinine and urea nitrogen levels, inhibit the activation of p53, reduce the level of ROS in renal tissue, block the activation of the mitochondrial and endoplasmic reticulum apoptosis pathways, and reduce the inflammatory response; however, these natural antioxidants have a weak antioxidant capacity and should only be used as dietary supplements. These compounds have been proved to not have renal protective properties. Furthermore, sodium thiosulfate can prevent DDP-induced nephrotoxicity and inhibits the antitumor effect of DDP, limiting its application. Thus, there remains a need for a specific drug for protection against DDP-induced nephrotoxicity.

Compound XH-003, an ARS-protective drug, was independently developed by our laboratory with 100% intellectual property rights. XH-003 is currently in a preclinical study, and the results show that XH-003 not only retains the strong antioxidant ability of amifostine but also overcomes the drawbacks of the drug. XH-003 can be administered orally, has a bioavailability of up to 42%, and reduces the toxic effects of amifostine. The properties of XH-003 as an ARS-protective agent have been shown to far exceed those of amifostine. Therefore, the aim of this study is to explore the potential of XH-003 against DDP-induced nephrotoxicity and to not only broaden the indications of XH-003 but also benefit more patients with cancer and improve their quality of life.

## 2. Concise Methods

### 2.1. Animals

Female SD rats (200–300 g) were used in all acute nephrotoxicity experiments. The animals were purchased from SPF Biotechnology Co. Ltd. (Beijing) and were bred in a certified animal facility at the Institute of Radiation Medicine (IRM) of the Chinese Academy of Medical Sciences (CAMS).

### 2.2. Ethics Approval and Consent to Participate

All experimental procedures were carried out in accordance with the NIH Guidelines for the Care and Use of Laboratory Animals and were approved by the Institutional Animal Care and Use Committee of the IRM, CAMS (permit number: 2017053). The animals were cared for in accordance with the guidelines of the National Animal Welfare Law of China.

### 2.3. DDP Dose Screening

A total of 12 female SD rats (230–260 g) were divided into four groups (*n* = 3 each), including a control group and three DDP treatment groups (5, 7.5, and 10 mg/kg, respectively). DDP was administered as a single intraperitoneal (i.p.) injection. The rats were monitored twice daily and weighted once a day, with their survival and behavior recorded. The experiment was terminated after 3 days, and all the rats were anesthetized and sacrificed. Blood samples were obtained, and sera were separated. Serum creatinine and urea were calculated using the SMT-100V portable automatic animal biochemical analyzer.

### 2.4. Determination of XH-003 Administration Time

A total of 15 female SD rats (230–260 g) were divided into five groups (*n* = 3 each), including a control group, DDP (5 mg/kg) group, XH-003 (800 mg/kg) group, and DDP (5 mg/kg)+XH-003 (800 mg/kg) groups A and B. Group A received a single PO dose of XH-003 (800 mg/kg) 30 min before DDP administration, and group B received a single PO dose of XH-003 (800 mg/kg) 4 h before DDP administration. On day 3, all the animals were anesthetized and sacrificed; blood samples were obtained, and sera were separated. Serum creatinine and urea were measured according to the abovementioned method.

### 2.5. Comparison of Chemoprotective Effects of XH-003 and Amifostine in Chemotherapy

A total of 18 female SD rats (230–260 g) were divided into six groups (*n* = 3 each), including a control group, DDP (5 mg/kg) group, XH-003 (800 mg/kg) group, amifostine (200 mg/kg) group, DDP (5 mg/kg)+XH-003 (800 mg/kg) group, and DDP (5 mg/kg)+amifostine (200 mg/kg) group. The DDP+XH-003 group received a single PO dose of XH-003 (800 mg/kg) 30 min before DDP administration, and the DDP+amifostine group received a single 200 mg/kg i.p. dose of amifostine 30 min before DDP administration. The rats were monitored twice daily and weighted once a day. The experiment was terminated after 3 days, and all the rats were anesthetized and sacrificed. Blood samples were obtained. The right kidney was fixed in 4% neutral formaldehyde for histological examination, and the left kidney was frozen in liquid nitrogen and stored at −80°C until analysis.

### 2.6. Kidney Function Tests

Serum samples were collected by centrifugation at 4,000 rpm for 10 min. Serum creatinine and urea nitrogen levels were measured according to the abovementioned method.

### 2.7. Hematological Evaluation

Blood was obtained from the rats via the orbital sinus and was collected in a micropipette coated with K_3_EDTA. The blood parameters measured included white blood cells (WBCs), red blood cells, platelets (PLTs), hemoglobin, monocytes (MOs), and basophils (BAs). Cells were counted using a Celltac E hemocytometer (Nihon Kohden, Japan).

### 2.8. Malondialdehyde (MDA) Assay

The MDA content was measured in kidney tissue using an MDA detection kit (No. A003-1; Nanjing Jiancheng Bioengineering Institute, Nanjing, China), according to the manufacturer's instructions.

### 2.9. T-SOD, GSH-Px, and CAT Assays

The activities of T-SOD, GSH-Px, and CAT in rat kidney tissues were tested using commercial kits (catalog numbers: A-001-1, A005-1, and A007-2-1, respectively), as specified by the manufacturer (Nanjing Jiancheng Bioengineering Institute).

### 2.10. Hematoxylin and Eosin (HE) Staining

The samples of renal tissue fixed in 4% paraformaldehyde were embedded in paraffin and cut into 5 *μ*m sections using a paraffin section machine. Subsequently, the sections were dewaxed with xylene and absolute ethanol to water, then stained with HE (71014460; Shanghai Jingke Chemical Technology Co., Ltd.) and analyzed under a microscope.

### 2.11. Terminal Deoxynucleotidyl Transferase-Mediated dUTP Nick-End Labeling (TUNEL) Staining

The 5 *μ*M tissue sections were treated according to the protocol for a TUNEL kit (11684817910; Roche). Analysis was performed under a microscope.

### 2.12. Western Blotting

Proteins were extracted from renal tissue using an ice-cold lysis buffer, and the protein concentrations were quantified using a BCA assay kit (PC0020; Solarbio). SDS-PAGE was used to separate equal amounts of protein. The blocked membrane was incubated using anti-Bax (1 : 500; WL01637, WanleiBio), anti-P-p53 (1 : 2,000; ab1431, Abcam), anti-PUMA (1 : 3,000; ab9643, Abcam), anti-caspase-3 (1 : 2,000; 9661, CST), anti-Nrf2 (1 : 500; ab89443, Abcam), anti-HO-1 (1 : 500; WL01637, WanleiBio), anti-OCT2 (1 : 500; ab243153, Abcam), anti-MRP2 (1 : 500; ab203397, Abcam), and anti-*β*-actin (1 : 1,000; WL01372, WanleiBio) antibodies overnight at 4°C. Then, the membranes were incubated with a suitable horseradish peroxidase-conjugated secondary antibody for 60 min at room temperature. Finally, an ECL western blotting substrate was used to visualize the immunoblots.

### 2.13. Platinum Uptake Assay

To evaluate the effect of XH-003 on the uptake of DDP by renal tissue, we examined the platinum (Pt) content in renal tissue using inductively coupled plasma mass spectrometry. In brief, tissue samples were lyophilized using a freeze dryer system. The lyophilized samples were digested with 0.2 mL of high-purity nitric acid at room temperature for 4 h, then diluted with 2 mL of deionized water and centrifuged at 6,000 rpm for 15 min. Finally, a 0.2 mL aliquot was transferred to an inductively coupled plasma mass spectrometry autosampler tube to determine the Pt content (*μ*g/g tissue) at each time point.

### 2.14. H358 *In Vivo* Tumor Model

H385 cells were cultured in Dulbecco's modified Eagle medium containing 10% fetal bovine serum. After reaching the exponential growth stage, cells were resuspended in phosphate-buffered saline and mixed (1 : 1) with Matrigel for subcutaneous inoculation into BALB/c nude mice. Fifty female mice were inoculated with 5 × 10^6^ H358 cells. When the average tumor volume reached approximately 200 mm^3^, the mice were randomly divided into the following four groups (*n* = 6 each): control group, XH-003 (500 mg/kg) single-dose group, DDP (10 mg/kg) single-dose group, and 500 mg/kg XH-003+10 mg/kg DDP group. XH-003 was administered orally once a week for 2 weeks, the first administration of XH-003 was 30 min before DDP administration, and the second administration of XH-003 was 8 h after the first administration. DDP was administered by tail vein injection once a week for 2 weeks. The therapeutic effect was evaluated based on the relative tumor inhibition rate. The results of tumor volume were expressed by mean ± standard error of mean. All data were analyzed with Prism 6.0. *p* < 0.05 was considered statistically significant.

## 3. Results

### 3.1. Optimal Dose of DDP for Building a Renal Injury Model

**Figure 1:**
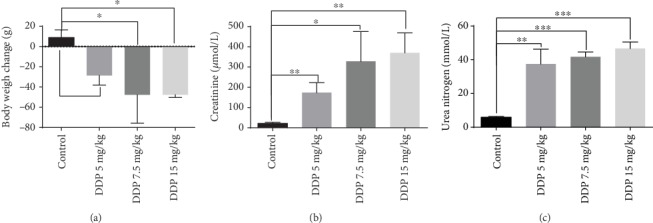
Changes in body weight (a), creatinine (b), and urea nitrogen (c) in rats after treatment with DDP (5–15 mg/kg). ^∗^*p* < 0.05, ^∗∗^*p* < 0.01, and ^∗∗∗^*p* < 0.001.

A single high dose of DDP, ranging from 5 to 15 mg/kg, is widely used to build renal injury models in rats [40]. However, differences in animal species, sources, animal room environments, etc., result in different optimal DDP doses for modeling and in different effects of renoprotective agents. To establish a rat renal injury model suitable for our study, three doses of DDP were tested, and the results are shown in Figure 1 and Supplementary Table S1. All rats in the treatment groups lost weight, whereas those in the control group gained weight (Figure 1(a)). Meanwhile, the levels of creatinine and urea nitrogen (Figures 1(b) and 1(c)) were significantly higher in rats treated with the different doses of DDP than in the control group. The mean serum creatinine and urea nitrogen levels were 21.67 ± 5.56 *μ*mol/L and 5.86 ± 0.53 mmol/L in the control group and increased to 173.53 ± 49.53 *μ*mol/L and 37.45 ± 8.85 mmol/L in the 5 mg/kg DDP group (*p* < 0.05), 328 ± 104.5 *μ*mol/L and 41.8 ± 1.91 mmol/L in the 7.5 mg/kg DDP group (*p* < 0.05), and 370.8 ± 69.3 *μ*mol/L and 46.69 ± 2.69 mmol/L in the 15 mg/kg DDP group (*p* < 0.05), respectively. Notably, one rat died in each 7.5 and 15 mg/kg DDP group, which was attributed to DDP intolerance. Considering these factors, the optimal single dose of DDP of 5 mg/kg was applied to study the protective effect of XH-003 against DDP-induced nephrotoxicity.

### 3.2. Optimal Time of XH-003 Administration prior to DDP Administration

**Figure 2:**
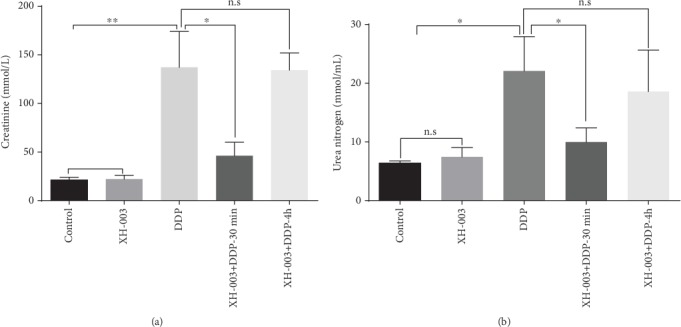
Effects of the time of administration of XH-003 on DDP-induced increases in serum creatinine (a) and urea nitrogen (b) levels. ^∗^*p* < 0.05, ^∗∗^*p* < 0.01, and ^∗∗∗^*p* < 0.001. n.s: no difference.

The most optimum administration time for XH-003 was 4 h before irradiation, and its half-life was 6.65 h. However, it remains unknown whether XH-003 would show a chemoprotective effect at this time of administration. Therefore, two times of administration were tested, 30 min and 4 h prior to DDP administration. The results are shown in Figure 2 and Supplementary Table S2. Compared with those in the control group, the levels of creatinine and urea nitrogen in the DDP group were significantly higher: 7.8 (159.3 ± 21.64 vs. 20.43 ± 3.82 *μ*mol/L; *p* < 0.01) and 4.7 (28.87 ± 6.36 vs. 6.09 ± 0.49 mmol/L; *p* < 0.05) times higher, respectively. Interestingly, compared with those in the DDP group, the levels of creatinine (87.17 ± 14.10 *μ*mol/L; *p* > 0.05) and urea nitrogen (15.51 ± 5.36 mmol/L; *p* > 0.05) slightly improved upon administration of XH-003 4 h prior to DDP administration, with no statistically significant differences. However, the administration of XH-003 30 min prior to DDP administration significantly decreased the levels of creatinine (46.17 ± 13.85 *μ*mol/L; *p* < 0.05) and urea nitrogen (10.91 ± 4.04 mmol/L; *p* < 0.05). In addition, compared with the control group, the XH-003-alone treatment group did not show any differences in the levels of creatinine (19.77 ± 1.90 *μ*mol/L; *p* > 0.05) and urea nitrogen (6.41 ± 0.7 mmol/L; *p* > 0.05). Therefore, administration of XH-003 30 min prior to DDP administration could significantly improve DDP-induced nephrotoxicity and play a chemoprotective role.

### 3.3. Renal Protection Effects of XH-003 Are Similar to Those of Amifostine

**Figure 3:**
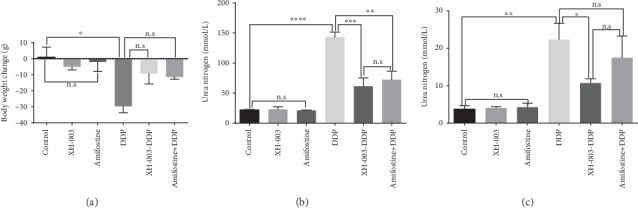
Comparison of renal protective effects between XH-003 and amifostine. (a) Changes in body weight in each group of rats. (b) Changes of serum creatinine level. (c) Changes of urea nitrogen in the serum. ^∗^*p* < 0.05, ^∗∗^*p* < 0.01, and ^∗∗∗^*p* < 0.001. n.s: no difference.

The primary purpose of designing XH-003 was to retain the efficacy of amifostine and overcome its drawbacks, such as nonoral administration, a short half-life, and strong side effects. As an ARS-protective agent, XH-003 has achieved this goal. However, it remains unknown whether the efficacy of XH-003, as a chemoprotective agent, is similar to that of amifostine. Therefore, we compared the protective effects of XH-003 and amifostine under the same experimental conditions. The results are shown in Figure 3 and Supplementary Table S3. The significant increases in serum creatinine (143.15 ± 8.25 vs. 61.39 ± 13.80 *μ*mol/L; *p* < 0.001) and urea nitrogen (22.33 ± 4.33 vs. 10.07 ± 1.17 mmol/L; *p* < 0.05), caused by DDP, were prevented in the XH-003+DDP group. Although amifostine significantly decreased creatinine (143.15 ± 8.25 vs. 72.14 ± 14.17 *μ*mol/L; *p* < 0.01) and urea nitrogen (22.33 ± 4.33 vs. 17.50 ± 5.16 mmol/L; *p* > 0.05), the decrease in the latter was not statistically significant. In addition, DDP-induced severe weight loss was alleviated by XH-003 and amifostine (Figure 3). It should be noted that there were no significant differences in the protective effects (61.39 ± 13.80 vs. 72.14 ± 14.17 *μ*mol/L for creatinine, *p* > 0.05; 10.07 ± 1.17 vs. 17.50 ± 5.16 mmol/L for urea nitrogen, *p* > 0.05; data not shown) of XH-003 and amifostine against DDP-induced nephrotoxicity. Thus, XH-003 retained the advantages of amifostine in protection against nephrotoxicity.

### 3.4. XH-003 Alleviates DDP-Induced Abnormalities in Peripheral Blood

**Figure 4:**
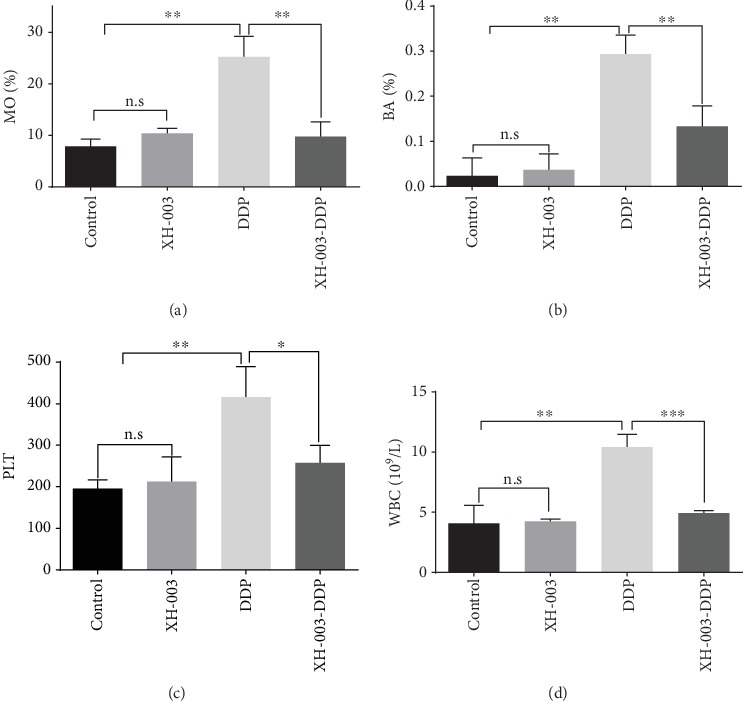
Effects of XH-003 on DDP-induced peripheral blood abnormalities, including (a) MO%, (b) BA%, (c) PLT counts, and (d) WBC counts. ^∗^*p* < 0.05, ^∗∗^*p* < 0.01, and ^∗∗∗^*p* < 0.001. n.s: no difference.

The evaluation of peripheral blood (Figure 4 and Supplementary Table S4) showed that in the DDP group, MO% (25.23 ± 4.03 vs. 7.85 ± 1.59; *p* < 0.01), BA% (0.29 ± 0.04 vs. 0.02 ± 0.04; *p* < 0.01), PLTs (416.33 ± 74.14 vs. 195.67 ± 19.66 × 10^9^/L; *p* < 0.01), and WBCs (10.62 ± 1.02 vs. 4.08 ± 1.49 × 10^9^/L; *p* < 0.01) significantly increased compared with their levels in the control group, suggesting that DDP induced inflammatory responses. However, XH-003 significantly improved DDP-induced levels of MO% (9.76 ± 2.88 vs. 25.23 ± 4.03; *p* < 0.01), BA% (0.13 ± 0.04 vs. 0.29 ± 0.04; p < 0.01), PLTs (257.33 ± 35.11 vs. 416.33 ± 74.14 × 10^9^/L; *p* < 0.05), and WBCs (4.93 ± 0.21 vs. 10.62 ± 1.02 × 10^9^/L; *p* < 0.001). There were no changes in other peripheral blood indexes (data not shown).

### 3.5. XH-003 Improves DDP-Induced Renal Inflammatory Infiltration

**Figure 5:**
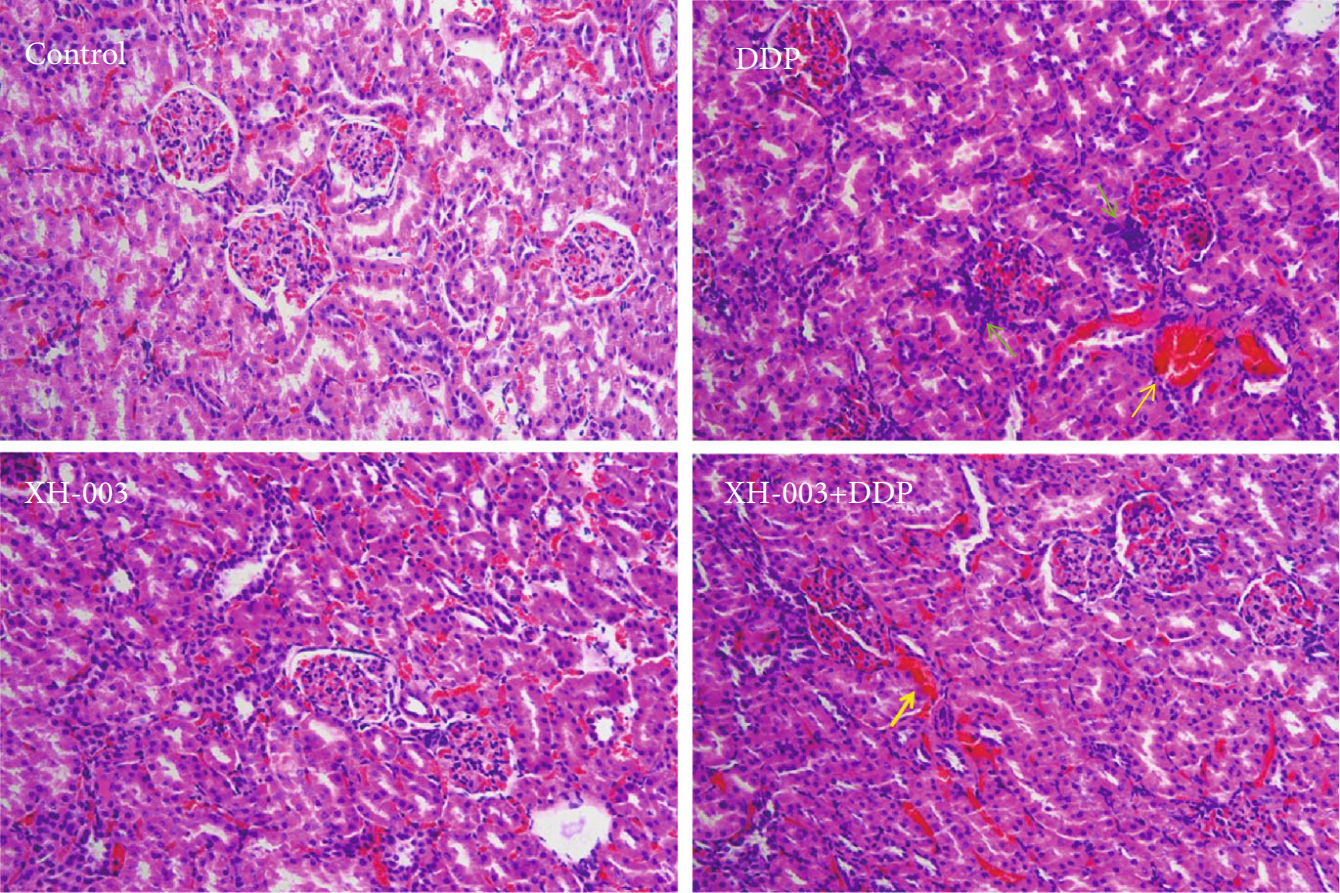
Effects of XH-003 on DDP-induced renal inflammation. Green arrows indicate inflammation, and yellow arrows indicate interstitial hyperemia.

Given the results of the peripheral blood evaluation, HE staining was used to observe whether DDP caused the inflammatory response in renal tissue and whether XH-003 could alleviate this effect (Figure 5). In the DDP group, the glomerular structure was clear, with no obvious lobular atrophy of the glomerulus. However, vascular congestion was observed in the stroma, and inflammatory cell infiltration was observed in the tissue. Meanwhile, XH-003 alleviated this inflammatory reaction, showing significant protection to the kidneys of the rats challenged with DDP.

### 3.6. XH-003 Relieves Oxidative Stress in the Kidneys of DDP-Treated Rats

**Figure 6:**
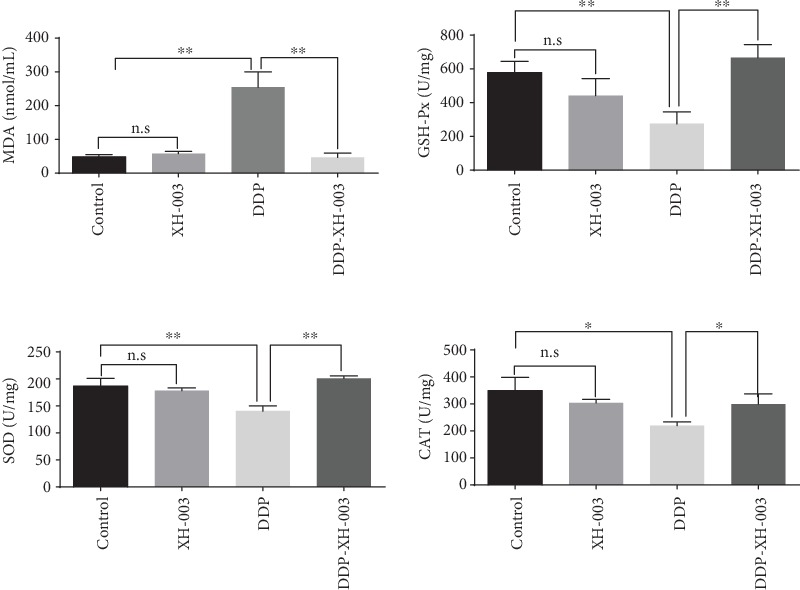
Effects of XH-003 on DDP-induced oxidative stress, based on the levels of MDA, GSH-Px, SOD, and CAT. ^∗^*p* < 0.05, ^∗∗^*p* < 0.01, and ^∗∗∗^*p* < 0.001. n.s: no difference.

Oxidative stress influences DDP-induced renal injury. The increase of MDA level and the decrease of antioxidant enzyme level are observed in DDP-induced renal injury. Next, we examined whether XH-003 could attenuate oxidative stress in the kidneys of DDP-treated rats. The results are shown in Figure 6 and Supplementary Table S5. DDP induced a significant increase in the MDA level (254.98 ± 44.82 vs. 47.71 ± 7.27 nmol/mL; *p* < 0.01) and decreased the levels of the antioxidant enzymes SOD (140.43 ± 9.45 vs. 186.80 ± 13.97 U/mg; p < 0.01), GSH-Px (274.69 ± 70.82 vs. 578.41 ± 66.91 U/mg; p < 0.01), and CAT (219.70 ± 14.32 vs. 349.14 ± 48.92 U/g; p < 0.05) in renal tissue compared with those in the control, respectively. Meanwhile, compared with DDP group, XH-003 significantly reduced the MDA level (46.43 ± 12.63 vs. 254.98 ± 44.82 U/mg; p < 0.01) and increased the antioxidant enzymes SOD (201.02 ± 4.03 vs. 140.43 ± 9.45 U/mg; p < 0.001), GSH-Px (666.91 ± 76.82 vs. 274.69 ± 70.82 U/mg; p < 0.01), and CAT (299.72 ± 37.52 vs. 219.70 ± 14.32 U/g; p < 0.05). These results indicated that XH-003 effectively alleviated the DDP-induced oxidative stress.

### 3.7. XH-003 Inhibits DDP-Induced Renal Apoptosis

**Figure 7:**
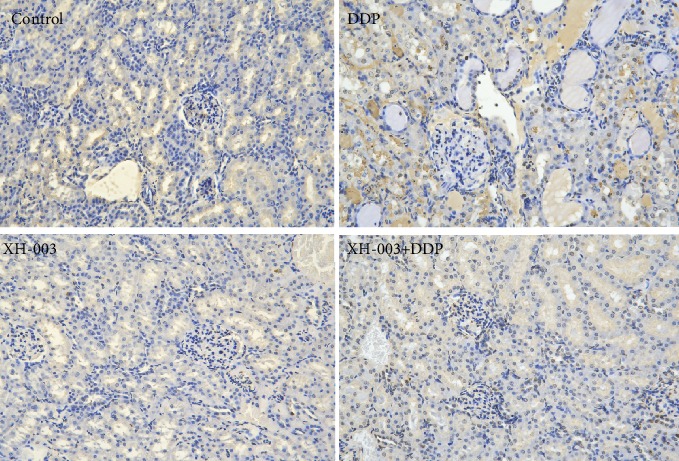
Effect of XH-003 on DDP-induced renal apoptosis, which is indicated by brown TUNEL staining.

We used TUNEL staining to evaluate apoptosis *in vivo* (Figure 7). Compared with the control and XH-003-only groups, which showed no apoptosis, DDP induced apoptosis in renal tissue, which was alleviated by XH-003, showing a significant protective effect against DDP-induced renal injury.

### 3.8. XH-003 Prevents the DDP-Induced Activation of the Mitochondrial Apoptosis Pathway

**Figure 8:**
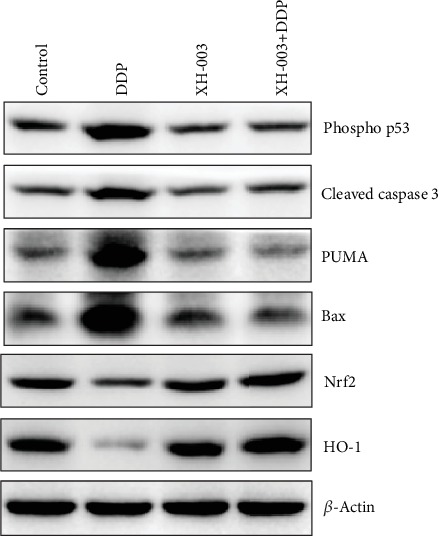
Effects of XH-003 on DDP-induced changes in the expression of mitochondrial apoptosis pathway- and antioxidation-related proteins.

To examine whether XH-003 prevents the activation of the DDP-induced apoptosis pathway, the expression levels of apoptosis pathway-related proteins were analyzed by western blotting (Figure 8). We found that XH-003 significantly reduced the expression levels of DDP-induced mitochondrial apoptosis pathway-related proteins such as cleaved caspase-3, PUMA, Bax, and phospho-p53. XH-003 also alleviated the DDP-induced decreases in the expression of the antioxidation-related proteins Nrf2 and HO-1.

### 3.9. XH-003 Reduces the Content of DDP in Renal Tissue

**Figure 9:**
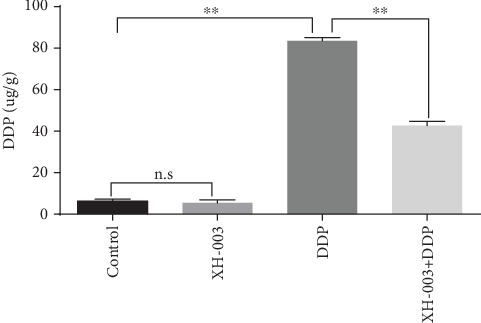
Effect of XH-003 on DDP accumulation in renal tissue.

To examine whether the renal protection effects of XH-003 are because of a decrease of DDP in kidney tissue, Pt levels were determined 3 days after DDP administration. The results are shown in Figure 9 and Supplementary Table S6. In the XH-003+DDP group, the level of Pt (42.58 ± 2.13 *μ*g/g) was half of that in the DDP group (83.46 ± 1.57 *μ*g/g; p < 0.001), indicating that XH-003 significantly reduced the level of Pt in renal tissue.

### 3.10. XH-003 Affects the Uptake and Excretion of DDP from Renal Tissue

**Figure 10:**
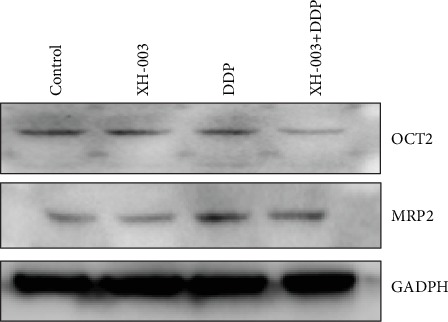
Effects of XH-003 on the expression of proteins related to the uptake and excretion of DDP.

We speculated that XH-003 might affect the uptake or excretion of DDP from renal tissue. OCT2 and MRP2 influence DDP uptake and excretion. Western blotting analysis was used to test whether XH-003 affected the expression of the OCT2 and MRP2 proteins (Figure 10). Compared with those in the DDP group, the expression level of OCT2 was decreased and that of MRP2 was increased by XH-003 treatment, indicating that XH-003 reduced the DDP uptake and increased its excretion from renal tissue. XH-003 alone could also increase the expression of MRP2.

### 3.11. XH-003 Does Not Affect the Antitumor Effect of DDP

**Figure 11:**
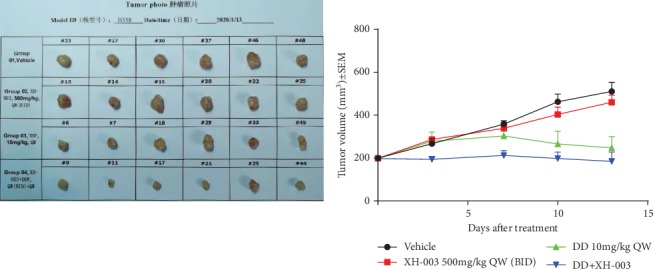
Effect of XH-003 on the antitumor efficacy of DDP.

In addition to its ability to reduce DDP-induced nephrotoxicity, it was very important to determine whether XH-003 attenuates the antitumor effect of DDP. The results of the H358 xenotransplantation experiment (Figure 11) showed that compared with those in the control (510.6 ± 103.8 mm^3^) and XH-003 (460.4 ± 80.2 mm^3^) groups, tumor volumes significantly declined in the DDP (247.3 ± 129.1 mm^3^) and DDP+XH-003 (184.4 ± 106.5 mm^3^) groups. Interestingly, the antitumor effect was significantly better (*p* < 0.001) in the XH-003+DDP group than in the DDP group, suggesting that XH-003 did not affect the antitumor activity of DDP but the combination of the two drugs might have had a synergistic effect.

## 4. Discussion

DDP, used as an antitumor agent since 50 years, has significantly contributed to the enormous advances in the field of oncology; however, there is no effective solution to the problem of DDP-induced nephrotoxicity. Oxidative stress and DNA damage play key roles in DDP-induced nephrotoxicity. Therefore, the inhibition of ROS by antioxidants is a potential approach to the treatment of DDP-induced nephrotoxicity.

In this study, we found that XH-003 also showed a significant protective effect against DDP-induced nephrotoxicity. XH-003 significantly reduced DDP-increased levels of creatinine and urea nitrogen in the plasma, which are well-known biomarkers of kidney function. Furthermore, XH-003 alleviated the DDP-induced abnormal changes in BA%, MO%, PLTs, and WBCs in the peripheral blood, suggesting that XH-003 could effectively improve DDP-induced inflammation. HE staining confirmed that XH-003 relieved DDP-induced renal tissue inflammation. Together with the results of TUNEL staining for apoptosis, the data confirmed that XH-003 had a good protective effect against DDP-induced nephrotoxicity.

Evidence obtained in recent years has indicated that DDP not only directly damages DNA through chelation but also induces renal damage by producing large amounts of ROS and indirectly damaging DNA. When DNA is damaged, it immediately activates the ataxia telangiectasia mutant protein, which can cause phosphorylation of p53. The role of p53, which is a well-recognized tumor suppressor protein, was elucidated in renal injury by Cummings and Schnellmann. Activated p53 can promote the expression of PUMA; disturb the interaction between the BCl-XL and Bax proteins; lead to the formation of the mitochondrial pore and the release of apoptotic proteins, such as cytochrome c; activate the caspase cascade; and induce apoptosis. Moreover, the increase in ROS can activate the endoplasmic reticulum pathway, followed by activation of the caspase-9/3 cascade and induction of apoptosis [41–43]. Our mechanistic study showed that XH-003 could effectively reduce the phosphorylation level of p53 and the expression of apoptosis-related proteins in the mitochondrial pathway, including PUMA, Bax, and cleaved caspase-3.

Oxidative stress in a physiological state can promote the dissociation of Nrf2 and keap-1 and Nrf2 translocation into the nucleus. Nrf2 is combined with ARE through MAF and activates the expression of downstream antioxidant genes, promoting the expression of antioxidant proteins, such as HO-1, SOD, and CAT [44, 45]. When DDP enters renal tissue, it induces excessive oxidative stress, which leads to excessive consumption of Nrf2, blocked expression of antioxidant proteins, and reduced antioxidant capacity. In this study, we found that XH-003 prevented DDP-induced inhibition of Nrf2 and HO-1 protein expression. Meanwhile, XH-003 significantly reduced the increase in MDA and alleviated DDP-induced decreases in antioxidant enzyme activities, indicating that XH-003 can effectively improve the DDP-induced oxidative stress in renal tissue.

DDP is easily accumulated, excreted, and metabolized in the kidney. Therefore, reducing the concentration of DDP in the kidney is an important strategy for kidney protection. Interestingly, in this study, XH-003 significantly reduced the DDP concentration in renal tissue, resulting in decreased nephrotoxicity. A previous study has shown that the uptake of DDP by renal tubular cells involves OCT2 and CTR1 and the efflux involves the MRP2 and MRP4 transporters [46]. Our results showed that XH-003 reduced the concentration of DDP in renal tissue by affecting the expression of the DDP transport- and excretion-related proteins OCT2 and MRP2, thereby playing a chemoprotective role.

XH-003 reduced the concentration of DDP in renal tissue, downregulated the expression of apoptosis-related proteins in the mitochondrial pathway, and protected normal renal tissue; however, it is unknown whether it also protected tumor tissue. The XH-003 dose used in this study was not effective in significantly reducing the antitumor effect of DDP and may have had a synergistic effect with DDP. Some antitumor drugs, such as metal drugs and DNA-damaging drugs, whose primary mechanism involves the activation of the apoptosis pathway and induction of cell death through excessive ROS production, can kill tumor cells by increasing ROS levels. However, there are some drugs that cause antitumor effects by removing ROS from tumor cells; these include vitamins C and E, whose primary mechanism targets the ability of ROS to promote tumor development via the oxidation of specific chemical groups in cells. This oxidation reaction can induce gene mutations and activate related biochemical pathways, thus promoting cell proliferation and tumorigenic transformation. Therefore, as a powerful antioxidant, XH-003 may also have potential application in the field of antitumor therapy.

Although current studies have shown that XH-003 has a protective effect on DDP-induced nephrotoxicity, there are some limitations. For example, in the experimental condition section, the selection of animal species and feeding conditions in each laboratory have an influence on the dosage of cisplatin, and there is no standardized modeling method, which can lead to variations in results. Although the experiment has been repeated many times, the number of rats in each group is small, which may affect the accuracy. In addition, the renal function index of this experiment evaluated creatinine and urea nitrogen, but did not evaluate other important indexes such as glomerular filtration rate and serum uric acid. Most importantly, the existing animal models can not completely simulate the situation of real clinical patients, so we need to explore new animal models to meet the clinical needs. Therefore, in the follow-up research, it is necessary to improve the experimental conditions and animal model, so that the preclinical research results can better reflect the situation of clinical patients.

## 5. Conclusion

XH-003 significantly reduced the accumulation of DDP in renal tissue, downregulated the expression of mitochondrial apoptosis pathway-related proteins, and alleviated oxidative stress and inflammation, thus providing renal protection without affecting the antitumor activity of DDP. Based on these results, chemoprotection can be considered an extended indication of XH-003.

## Data Availability

The data used to support the findings of this study are included within the article.

## Supplementary Materials

Supplementary Materials Table S1: the analysis of creatinine and urea nitrogen in rats (mean ± standard deviation). Table S2: the analysis of creatinine and urea nitrogen in rats treated with different time of XH-003 (mean ± standard deviation). Table S3: comparison of renal protective effects between XH-003 and amifostine (mean ± standard deviation). Table S4: the effect of XH-003 on DDP induced peripheral blood abnormality (mean ± standard deviation). Table S5: effect of XH-003 on biological indexes of oxidative stress (mean ± standard deviation). Table S6: evaluation the effect of XH-003 on DDP accumulation in renal tissue.
