# Supplementary material for: Renoprotective Effects of a New Free Radical Scavenger, XH-003, against Cisplatin-Induced Nephrotoxicity
Source: Oxid Med Cell Longev. 2020 Apr 18;2020:9820168. doi: 10.1155/2020/9820168 (PMC7189338; doi:10.1155/2020/9820168)
Supplement: Supplementary Materials — Table S1: the analysis of creatinine and urea nitrogen in rats (mean ± standard deviation). Table S2: the analysis of creatinine and urea nitrogen in rats treated with different time of XH-003 (mean ± standard deviation). Table S3: comparison of renal protective effects between XH-003 and amifostine (mean ± standard deviation). Table S4: the effect of XH-003 on DDP induced peripheral blood abnormality (mean ± standard deviation). Table S5: effect of XH-003 on biological indexes of oxidative stress (mean ± standard deviation). Table S6: evaluation the effect of XH-003 on DDP accumulation in renal tissue. [file 9820168.f1.docx]

Supplementary material

**Ya-Hong Liu^#1^, Kui Li^1^, Hong-Qi Tian*^1^**

1. Tianjin Key Laboratory of Radiation Medicine and Molecular Nuclear Medicine, Institute of Radiation Medicine, Chinese Academy of Medical Sciences and Peking Union Medical College, No. 238, Baidi Road, Tianjin, China

*Corresponding author. Tianjin Key Laboratory of Radiation Medicine and Molecular Nuclear Medicine, Institute of Radiation Medicine, Chinese Academy of Medical Sciences and Peking Union Medical College, No. 238, Baidi Road, Tianjin, China

E-mail address:tianhongqi@irm-cams.ac.cn (Hong-Qi Tian)

Table S1 The analysis of creatinine and urea nitrogen in rats (mean ± standard deviation)

| Group | Creatinine (μmol/L) | P value | Urea nitrogen  (mmol/L) | P value |
| --- | --- | --- | --- | --- |
| Control | 21.67 ± 5.56 | - | 5.86 ± 0.53 | - |
| DDP 5 mg/kg | 173.53 ± 49.53** | 0.006 | 37.45 ± 8.85** | 0.004 |
| DDP 7.5 mg/kg | 328 ± 104.5* | 0.029 | 41.8 ± 1.91*** | 0.0002 |
| DDP 15 mg/kg | 370.8 ± 69.3** | 0.007 | 46.69 ± 2.69*** | 0.0003 |

*p<0.05, **p<0.01, ***p<0.001

Table S2 The analysis of creatinine and urea nitrogen in rats treated with different time of XH-003 (mean ± standard deviation)

| Group | Creatinine (μmol/L) | P value | Urea nitrogen  (mmol/L) | P value |
| --- | --- | --- | --- | --- |
| Control | 20.43±3.82 | - | 6.09±0.49 | - |
| XH-003 | 19.77±1.90 | 0.75 | 6.41±0.7 | 0.32 |
| DDP 5 mg/kg | 159.3±21.64** | 0.005 | 28.87±6.36* | 0.014 |
| XH-003+DDP -30 min | 46.17±13.85* | 0.015 | 10.91±4.04* | 0.03 |
| XH-003+DDP -4 h | 87.17±14.10 | 0.9 | 15.51±5.36 | 0.54 |

*p<0.05, **p<0.01, ***p<0.001

Table S3 Comparison of renal protective effects between XH-003 and amifostine (mean ± standard deviation)

| Group | Creatinine (μmol/L) | P value | Urea nitrogen  (mmol/L) | P value |
| --- | --- | --- | --- | --- |
| Control | 22.2 ± 0.80 | - | 3.57 ± 0.65 | - |
| XH-003 | 22.33 ± 4.32 | 0.74 | 4.02 ± 0.39 | 0.67 |
| Amifostine | 21.13 ± 0.74 | 0.16 | 4.72 ± 1.08 | 0.57 |
| DDP 5 mg/kg | 143.15 ± 8.25*** | <0.0001 | 22.33 ± 4.33** | 0.0019 |
| XH-003+DDP -30 min | 61.39 ± 13.80*** | 0.0009 | 10.07 ± 1.17* | 0.011 |
| Amifostine+DDP -30 min | 72.14 ± 14.17** | 0.0017 | 17.50 ± 5.16 | 0.3 |

*p<0.05, **p<0.01, ***p<0.001

Table S4 The effect of XH-003 on DDP induced peripheral blood abnormality (mean ± standard deviation)

|  | Control | XH-003 | DDP | XH-003+DDP |
| --- | --- | --- | --- | --- |
| MO% | 7.85±1.59 | 10.04±0.96 | 25.23±4.03** | 9.76±2.88** |
| P value | - | 0.076 | 0.0022 | 0.0056 |
| BA% | 0.02±0.04 | 0.04±0.04 | 0.29±0.04** | 0.13±0.04** |
| P value | - | 0.6885 | 0.0012 | 0.0088 |
| PLT 10^9^/L | 195.67±19.66 | 212.33±58.96 | 416.33±74.14** | 257.33±35.11* |
| P value | - | 0.6665 | 0.0076 | 0.0284 |
| WBC 10^9^/L | 4.08±1.49 | 4.23±0.28 | 10.62±1.02** | 4.93±0.21*** |
| P value | - | 0.87 | 0.004 | 0.0009 |

*p<0.05, **p<0.01, ***p<0.001

Table S5 Effect of XH-003 on Biological Indexes of oxidative stress (mean ± standard deviation)

|  | Control | XH-003 |  | DDP | XH-003+DDP |
| --- | --- | --- | --- | --- | --- |
| MDA（nmol/mL） | 47.71±7.27 | 57.62±6.64 |  | 254.98±44.82** | 46.43±12.63** |
| P value | - | 0.1564 |  | 0.0014 | 0.0015 |
| SOD  (U/mg) | 186.80±13.97 | 178.43±4.99 |  | 140.43±9.45** | 201.02±4.03*** |
| P value | - | 0.3838 |  | 0.0089 | 0.0006 |
| GSH-Px  (U/mg) | 578.41±66.91 | 441.85±101.62 |  | 274.69±70.82** | 666.91±76.82** |
| P value | - | 0.1231 |  | 0.0057 | 0.0029 |
| CAT  (U/g) | 349.14±48.92 | 304.42±12.58 |  | 219.70±14.32* | 299.72±37.52* |
| P value | - | 0.1999 |  | 0.0117 | 0.0260 |

*p<0.05, **p<0.01, ***p<0.001

Table S6 Evaluation the effect of XH-003 on DDP accumulation in renal tissue

|  | Control | XH-003 | CDDP | XH-003+CDDP |
| --- | --- | --- | --- | --- |
| DDP（ug/g） | 6.33±0.86 | 5.45±1.44 | 83.46±1.57*** | 42.58±2.13*** |
| P value | - | 0.4106 | <0.001 | <0.001 |

*p<0.05, **p<0.01, ***p<0.001
